# Normative data for dimensions of the frontal and infraorbital nerves

**DOI:** 10.1038/s41433-025-03679-4

**Published:** 2025-02-07

**Authors:** Jessica Y. Tong, Jeffrey Sung, Khizar Rana, WengOnn Chan, Alkis J. Psaltis, Dinesh Selva

**Affiliations:** 1 South Australian Institute of Ophthalmology, Royal Adelaide Hospital, Adelaide, SA Australia; 2 Discipline of Ophthalmology and Vision Sciences, University of Adelaide, Adelaide, SA Australia; 3 Faculty of Health and Medical Sciences, The University of Adelaide, Adelaide, SA Australia; 4 Department of Otolaryngology Head and Neck Surgery, Queen Elizabeth Hospital, Woodville, SA Australia; 5 Department of Surgery-Otolaryngology, Head and Neck Surgery University of Adelaide, Adelaide, SA Australia

**Keywords:** Anatomy, Eye diseases

## Abstract

**Objectives:**

To present a series of normative measurements for the width of the frontal and infraorbital nerve branches of V1 and V2, respectively.

**Methods:**

Cadaveric dissection study of 15 embalmed cadaver heads (30 orbits). The frontal nerve was excised en bloc from the superior orbital rim to the superior orbital fissure. Similarly, the infraorbital nerve was excised en bloc from the inferior orbital rim to the orbital apex. Measurements were recorded of the maximal width of the frontal nerve, infraorbital nerve within the orbital floor, and pterygopalatine segment of the maxillary nerve. Any value greater than 2 standard deviations (SD) above the mean value, was defined as nerve enlargement.

**Results:**

The mean transverse diameter of the frontal nerve was 2.27 ± 0.66 mm (1 SD). The mean transverse diameter of the infraorbital nerve branch, and the maxillary nerve within the pterygopalatine fossa, was 3.31 ± 0.68 mm (1 SD) and 3.59 ± 0.76 mm (1 SD), respectively. The upper limit of normal, defined as 2 SD above the mean value, for the widths of the frontal nerve, infraorbital nerve, and pterygopalatine segment of V2 widths were 3.59 mm, 4.67 mm, and 5.10 mm, respectively.

**Conclusions:**

The frontal and infraorbital nerves are implicated in various inflammatory and neoplastic orbital pathologies. Defining the normative data for width is important to compare with pathological states.

## Introduction

Enlargement of the frontal and infraorbital nerves is an important radiological sign. It is highly suggestive of malignant perineural spread or IgG4-related disease of the orbit, but can also be involved in other neoplastic or inflammatory conditions. However, ‘enlargement’ is a subjective term as there is currently no point of reference for normative dimensions of the frontal or infraorbital nerve. Radiologically, it is also difficult to accurately determine the maximal diameter of either nerve in normal cohorts given its small calibre.

To date, measurements of frontal and infraorbital nerve diameter have been reliant on cadaveric skull and 3D-CT radiographic studies. This data has focused on the diameter of the foramina or notches along the orbital rim, which only represents the dimensions of the supraorbital nerve and infraorbital nerve at their distal exit points. Clinically however, it would be more relevant to determine nerve width within the orbit, as any pathological process is seldom isolated to the orbital rim. Another limitation of these prior studies is that the supraorbital or infraorbital nerve measurements omitted the smaller branches exiting through accessory foramina. In fact, a study of South Indian human skulls found accessory foramina to be present in 66.25% of cases [1].

This study aims to address this gap in knowledge by providing data on the maximal width of the frontal nerve, infraorbital nerve and V2 within the pterygopalatine fossa. Cadaveric dissection studies may emulate the physiological dimensions of these nerves and provide useful points of reference in pathological states.

## Methods

A cadaveric dissection study was performed of 15 embalmed human heads (30 orbits). Red and blue coloured silicone was injected into the bilateral common carotid arteries and internal jugular veins respectively. The frontal nerve was excised en bloc from the superior orbital rim back to the superior orbital fissure via an eyelid crease approach. The infraorbital nerve branch extending from the infraorbital foramen back to the maxillary nerve at the foramen rotundum was excised, as previously described [2]. Following the nerves’ en bloc excision, the maximum transverse diameter of the frontal and infraorbital nerves within the orbit, and the maxillary nerve within the pterygopalatine fossa were visualised and measured. All measurements were recorded using a ruler with 1 mm increments and therefore a precision of 0.5 mm. The mean and standard deviation values were derived from these measurements, and the values represented to 2 decimal points. Ethics approval was provided by the institutional review board.

## Results

The measurements of the frontal and infraorbital nerves within the orbit, and maxillary nerve in the pterygopalatine fossa, are presented in Table 1. For ease of interpretation, 1 and 2 standard deviation (SD) data is included. The range of normative data was established as 2 standard deviations above and below the mean value. Any value greater than 2 standard deviations above the mean value, was defined as nerve enlargement.

**Table 1:** Measurements of the frontal nerve, infraorbital nerve and pterygopalatine segment of the maxillary nerve.

	Minimum (mm)	Maximum (mm)	Mean (mm)	1 SD (mm)	2 SD (mm)
Diameter of frontal nerve	1.00	3.50	2.27	0.66	1.32
Diameter of infraorbital nerve	2.00	4.50	3.31	0.68	1.36
Diameter of maxillary nerve (pterygopalatine fossa)	2.00	5.00	3.59	0.76	1.51

The mean diameter of the frontal nerve within the orbit was 2.27 mm ± 0.66 mm (1 SD). The mean diameter of the maxillary nerve in the pterygopalatine fossa was 3.59 mm ± 0.76 mm (1 SD) and its infraorbital nerve branch within the orbital floor was 3.31 mm ± 0.68 mm (1 SD). The upper limit of normal, defined as 2 SD above the mean value, for the widths of the frontal nerve, infraorbital nerve, and pterygopalatine segment of V2 widths were 3.59 mm, 4.67 mm, and 5.10 mm, respectively.

## Discussion

This cadaveric dissection study of 30 orbits presents the average measurements and the range of normative data for the intraorbital measurements of V1 and V2. In this study, the mean transverse diameter and upper limit of normal for the frontal nerve, infraorbital nerve, and the pterygopalatine segment of V2 widths were: 2.27 mm/3.59 mm, 3.31 mm/4.67 mm, and 3.59 mm/5.10 mm, respectively.

A multitude of cadaveric skull studies have elucidated the dimensions of the supraorbital notch or foramen. Naturally, a broad notch would have a much wider transverse diameter than a foramen, but some studies combined the measurements to derive an average value. Of these, the mean transverse diameter was recorded as 4.24–5.58 mm and the mean vertical diameter was 2.04–2.17 mm [1, 3–6]. In a study of adult skulls where measurements of the foramen or notch were separately analysed, the average transverse and vertical diameters of the ovoid supraorbital foramen were 3.78 mm and 2.02 mm, while the supraorbital notch was on average 5.7 mm wide [7]. In contrast, the measurements observed in 3D CT studies were considerably smaller. The mean diameter for the supraorbital foramen and supraorbital notch was 2.3–2.34 mm and 3.37–3.5 mm, respectively [8, 9]. There may have been a discrepancy between the measurements as the cadaveric studies combined notch and foramen diameter values, which may have skewed the mean to a higher value if there was a higher proportion of broad notches.

The frontal nerve diameter may also be inferred as a combination of the supraorbital and supratrochlear nerves. One cadaveric study obtained the measurements from their branching point of origin from the frontal nerve in 50 cadaveric heads [10]. The authors observed that in orbits with a distal frontal nerve branching point, the supraorbital and supratrochlear nerve diameters were 1.58 ± 0.24 mm and 1.09 ± 0.25 mm, respectively. In comparison, in orbits with a frontal nerve that branched proximally, the supraorbital and supratrochlear nerve widths were 1.54 ± 0.38 mm and 0.79 ± 0.18 mm, respectively. When data from the two anatomical variants was combined, the transverse diameter of the supraorbital nerve was 1.56 ± 0.30 mm and the supratrochlear nerve width was 0.95 ± 0.26 mm [10]. In our study, the mean width of the frontal nerve was 2.27 mm ± 0.66 mm, which approximates the addition of the supraorbital and supratrochlear widths obtained from this previous study. In a separate study, the diameters of the supraorbital and supratrochlear nerve and their respective smaller branches when present, were quantified at their exit points along the superior orbital rim [11]. The mean diameters obtained of the supraorbital nerve, its branch, the supratrochlear nerve and its branch, were 1.3 ± 0.2 mm, 0.9 ± 0.3 mm, 0.7 ± 0.1 mm and 0.6 ± 0.3 mm, respectively. The total value derived here approximated 3.5 mm, but this may have been due to the heterogeneous branching patterns, as the smaller nerve branches are not uniformly present.

The dimensions of the infraorbital foramen were also analysed across a number of cadaveric adult skull studies. The average horizontal diameter was 4.24–4.50 mm in women and 4.43–4.89 mm in men, or an overall average of 4.8 mm [4, 5, 12]. In another study of 79 adult skulls, the mean width was 3.7 mm, though the range of values was broad extending from 1.8 mm to 6.9 mm [6]. It was observed that 15% of cadavers had several infraorbital foramina, although the dimensions of any accessory openings were omitted and only the widest or primary foramen was included [12]. In comparison, in this study, the width of V2 within the pterygopalatine fossa was 3.59 mm ± 0.76 mm and its infraorbital nerve branch was 3.31 mm ± 0.68 mm. Although it would be expected that the maximal diameter of the infraorbital nerve within the orbital floor would be greater than the transverse axis of the infraorbital foramen, it may be that the latter structure is wider to account for the infraorbital artery accompanying the nerve. In our dissections, the infraorbital artery was frequently found to lie medial to the infraorbital nerve, but to improve the accuracy of our data, the artery was excluded from measurements of the nerve width.

Orbital nerve enlargement has been described in perineural spread from malignancy, orbital inflammatory disease and lymphoproliferative disorders. Neoplastic perineural spread, particularly in the context of squamous cell carcinoma, has a predilection for the trigeminal and facial nerves [13]. Within the orbit, the frontal nerve, infraorbital nerve and their respective branches can be involved [14]. Frontal nerve enlargement has been reported in cases of reactive lymphoid hyperplasia and various orbital inflammatory disorders, including adult-onset xanthogranuloma and idiopathic granulomatous orbital inflammation [15–18]. IgG4-related ophthalmic disease (IgG4-ROD) most commonly causes V2 enlargement but has also been observed in the frontal nerve and its supraorbital nerve branches [19, 20]. In a study of 15 patients with IgG4-related ROD, infraorbital nerve enlargement was found to have a 53% sensitivity and 100% specificity, and its presence was marked by the infraorbital canal being directly contiguous with an inflammatory mass [21]. Neurolymphomatosis, or lymphomatous involvement of the peripheral nervous system, is a rare phenomenon. Within the orbit, V2 enlargement due to lymphoma has been observed along its pathway from the cavernous sinus to the foramen rotundum, pterygopalatine fossa and inferior orbital fissure, with increasing frequency distally [22]. Other rarer causes of infraorbital nerve expansion are reactive lymphoid hyperplasia, sarcoidosis, neuronal tumours including neurofibromatosis and schwannoma, and aspergillosis [18, 23–27].

A limitation of this study is that the measurements were taken of nerves in embalmed heads where inevitably there would be a degree of tissue shrinkage. In a study of embalmed sheep tissue, there was a median shrinkage of 16.7% in width after 96 h of fixation, although it was noted that there was no discernible difference in tissue shrinkage after 24 h of tissue submersion [28]. In the range of 2–3 mm, which approximates the data obtained in this study, a 16.7% shrinkage rate following fixation would account for 0.3–0.5 mm of error. Radiologically, this difference would not be discernible.

## Conclusion

The frontal nerve and V2 within the pterygopalatine fossa and its infraorbital branch, are sensory nerves within the orbit that can all be involved by a myriad of inflammatory and neoplastic processes. Currently subjective measures of nerve enlargement predominate. The cadaveric data presented in this study provides a normative frame of reference to enable more accurate radiological interpretations.

## Summary

### What was known before

- The frontal nerve and infraorbital nerve are involved and enlarged in various inflammatory and neoplastic pathologies.
- Previous frontal and infraorbital nerve dimensions have been based on radiological or human skull studies of the respective foramina or notches.

### What this study adds

- A cadaveric dissection study provides a good approximation of in vivo structures and their dimensions.
- The mean transverse diameter of the frontal nerve, infraorbital nerve and pterygopalatine segment of V2 was: 2.27 mm, 3.31 mm and 3.59 mm, respectively.
- Nerve enlargement was defined as 2 standard deviations above the mean value, such that the upper limit of normal of the frontal nerve, infraorbital nerve and pterygopalatine segment of V2 was: 3.59 mm, 4.67 mm and 5.10 mm, respectively.

## Supplementary information

Eye Reporting Checklist
